# The role of nutritional and inflammatory markers in predicting postoperative complications after esophagectomy for esophageal squamous cell carcinoma: mechanisms, clinical applications, and future perspectives

**DOI:** 10.3389/fsurg.2025.1671783

**Published:** 2025-10-10

**Authors:** Junjie Shi, Sujie Tang, Cheng Shen, Dafu Xu, Wen Ze Tian, Zhiyun Xu

**Affiliations:** The Affiliated Huai'an No. 1 People’s Hospital of Nanjing Medical University, Huai'an, China

**Keywords:** esophageal squamous cell carcinoma, esophagectomy, postoperative complications, nutritional markers, inflammatory markers, prediction model

## Abstract

Esophageal squamous cell carcinoma (ESCC) is a prevalent malignancy with a high mortality rate, for which esophagectomy remains the cornerstone of curative treatment. However, this complex surgical procedure is associated with significant postoperative morbidity and mortality. Nutritional status and systemic inflammatory response are critically intertwined and play a pivotal role in the host's ability to withstand surgical stress and mount an effective recovery. This review aims to provide a comprehensive overview of the role of nutritional and inflammatory markers in predicting postoperative complications following esophagectomy for ESCC. We first elucidate the intricate biological mechanisms through which malnutrition and systemic inflammation compromise tissue repair, immune function, and overall surgical outcomes. We then systematically evaluate the predictive value of various individual markers, such as albumin, C-reactive protein (CRP), and neutrophil-to-lymphocyte ratio (NLR), as well as combined scoring systems like the Prognostic Nutritional Index (PNI) and the Glasgow Prognostic Score (GPS). The clinical application of these markers in preoperative risk stratification, guiding perioperative immunonutrition, and dynamic monitoring for early complication detection is thoroughly discussed. Finally, we highlight future perspectives, including the integration of novel biomarkers from metabolomics and proteomics, the application of artificial intelligence in building sophisticated prediction models, and the design of marker-guided precision intervention trials. A deeper understanding and smarter utilization of these readily available and cost-effective markers will pave the way for personalized perioperative management, ultimately improving the prognosis for patients with ESCC undergoing esophagectomy.

## 1 Introduction

### 1.1 Epidemiology and current treatment of esophageal squamous cell carcinoma (ESCC)

Esophageal carcinoma is the eighth most common malignancy and the sixth leading cause of cancer-related death worldwide, with significant geographical disparities in its incidence and mortality (1, 2). ESCC is the predominant histological subtype, particularly dominant in the “esophageal cancer belt” of East Asia, where it accounts for over 90% of esophageal cancer cases in China (3, 4). Despite considerable advancements in diagnostic techniques, neoadjuvant/adjuvant therapies, and surgical procedures, the overall prognosis for patients with ESCC remains suboptimal, with a 5-year survival rate hovering between 20% and 30% (5).

Esophagectomy with lymph node dissection stands as the core, potentially curative treatment modality for localized ESCC (6). The popularization of Minimally Invasive Esophagectomy (MIE) has somewhat reduced surgical trauma (7, 8). Nevertheless, esophagectomy itself remains one of the most traumatic and technically complex operations in digestive tract surgery, involving thoracic, abdominal, and sometimes cervical fields. The incidence of postoperative complications is persistently high, reported to be between 40% and 60% (9, 10).

### 1.2 The severity and classification of postoperative complications

Postoperative complications are a critical determinant of both short-term and long-term outcomes for ESCC patients. These complications not only increase patient suffering, prolong hospital stays, and escalate medical costs but can also lead to treatment-related mortality (11). Severe complications such as anastomotic leakage, chylothorax, and acute respiratory distress syndrome (ARDS) carry extremely high mortality rates (12, 13). Broadly, complications can be classified into two categories: surgical technique-related and systemic. The former includes anastomotic leakage, anastomotic stenosis, hemorrhage, and recurrent laryngeal nerve palsy; the latter is most commonly represented by pneumonia, but also includes thromboembolic events, cardiovascular events, and multiple organ dysfunction syndrome (MODS) (14, 15). The occurrence of these complications not only directly threatens life but may also delay the initiation of subsequent adjuvant therapies, thereby compromising long-term oncological control (16).

### 1.3 The central role of nutrition and inflammation in tumor progression and postoperative recovery

Patients with ESCC commonly suffer from malnutrition during their disease course. On one hand, symptoms such as dysphagia, anorexia, and cachexia caused by the tumor itself lead to inadequate intake (17, 18). On the other hand, cancer as a consumptive disease depletes the body's energy and protein reserves through its rapid proliferation and metabolic reprogramming. Malnutrition is not merely a consequence of tumor progression but also a significant negative factor affecting treatment tolerance and postoperative outcomes by suppressing immune function and weakening tissue repair capabilities (19).

Concurrently, the systemic inflammatory response is another core axis in the host's fight against the tumor. Cancer cells can induce a persistent, low-grade systemic inflammatory state by releasing various cytokines and chemokines, such as interleukin-6 (IL-6) and tumor necrosis factor-alpha (TNF-α) (20). This inflammatory microenvironment not only promotes tumor angiogenesis, invasion, and metastasis but also exacerbates the body's catabolism and immunosuppression (21). Surgical trauma itself, as a potent stressor, further triggers an acute, cascading inflammatory response, the so-called “second hit” (22). When a body already in a state of malnutrition and chronic inflammation encounters the immense trauma of surgery, its internal homeostasis is easily disrupted, leading to immune collapse and organ dysfunction, thus creating a “fertile ground” for the development of postoperative complications.

### 1.4 The necessity and clinical significance of predicting complications using biomarkers

Given the severity of postoperative complications, the precise identification of high-risk patients preoperatively and the implementation of targeted perioperative interventions have become key scientific questions for improving ESCC patient prognosis. Traditional risk assessment models, such as the American Society of Anesthesiologists (ASA) classification and the Charlson Comorbidity Index (CCI), while valuable, are relatively macroscopic and fail to fully capture the individualized nutritional and inflammatory status of the patient, thus having limited predictive efficacy (23).

In recent years, a series of blood-based biomarkers reflecting the body's nutritional reserves and systemic inflammation levels have garnered significant attention due to their convenience, low cost, and good reproducibility. These markers, such as serum albumin, prealbumin, C-reactive protein (CRP), and ratios derived from blood cell counts like the neutrophil-to-lymphocyte ratio (NLR) and platelet-to-lymphocyte ratio (PLR), have been shown to be closely associated with the prognosis and postoperative complications in patients with various solid tumors (24–26). Integrating these single or combined markers into predictive models holds the promise of achieving precise, dynamic, and individualized risk assessment for postoperative complications. This can provide a vital basis for clinical decision-making, such as intensifying preoperative nutritional support for high-risk patients, optimizing surgical timing, choosing more refined surgical methods, and conducting closer postoperative monitoring and early intervention (27).

### 1.5 Structure and purpose of this review

This review aims to systematically summarize the research from the last five years on the role of nutritional and inflammatory markers in predicting postoperative complications after esophagectomy for ESCC. We will first delve into the biological mechanisms by which malnutrition and systemic inflammation affect postoperative recovery. Second, we will detail the clinical evidence and predictive value of various single and composite markers. Subsequently, we will discuss how to integrate these markers into the practical management of perioperative patients. Finally, we will look forward to future directions in the field, including the exploration of novel biomarkers and the application of advanced technologies like artificial intelligence. Through this article, we hope to provide a comprehensive and in-depth reference for clinicians and researchers to promote biomarker-based precision perioperative management, ultimately improving the clinical outcomes of patients with ESCC.

## 2 Biological mechanisms underlying the impact of nutritional and inflammatory status on postoperative complications

**Figure 1:**
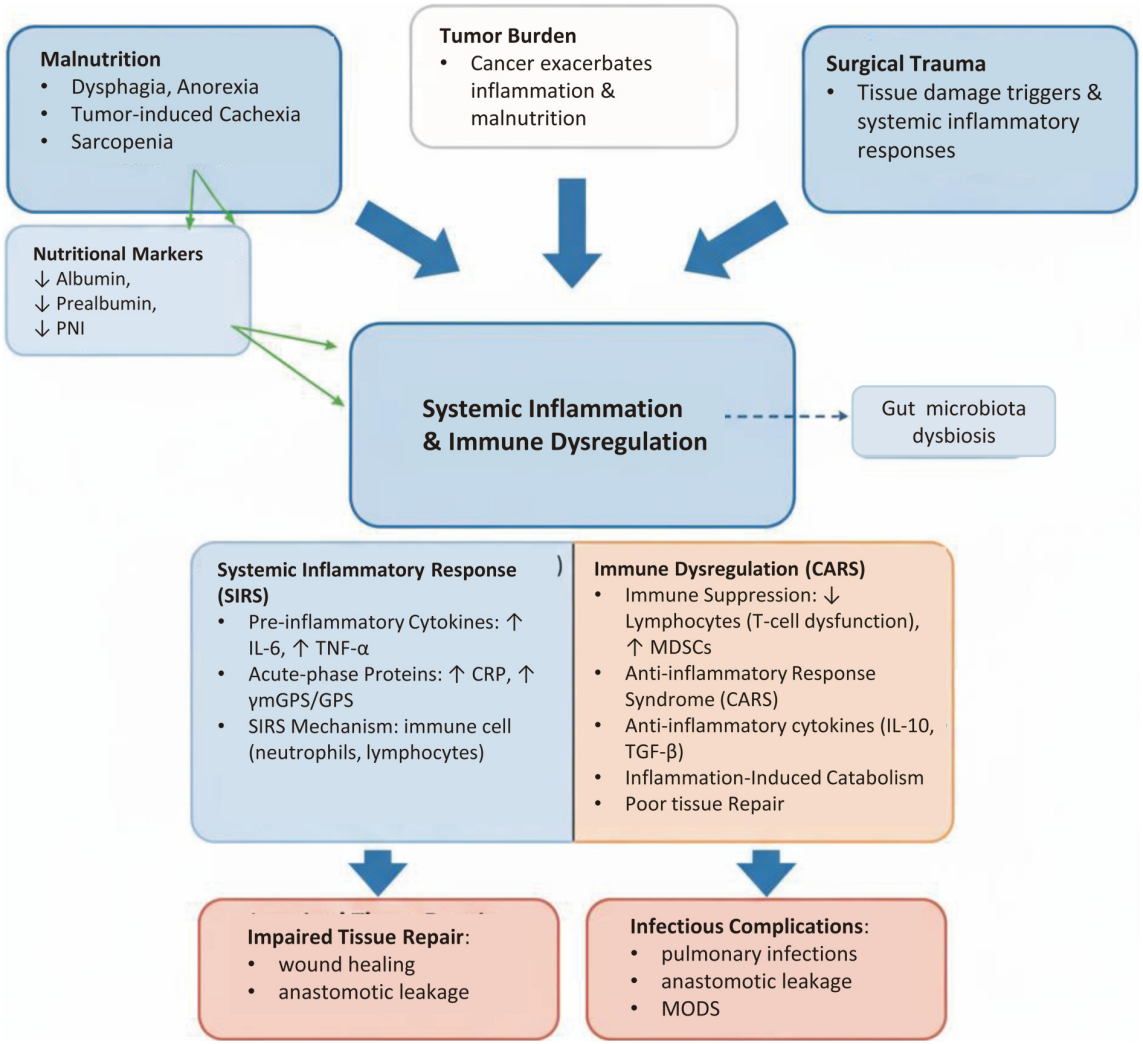
Pathophysiological triangle linking malnutrition, tumor burden, and surgical trauma to postoperative complications in ESCC. The diagram illustrates how malnutrition, tumor burden, and surgical trauma interact to create a favorable environment for postoperative complications in esophageal squamous cell carcinoma (ESCC). The central panel has been reorganized into clear bullet points, emphasizing the balance between Systemic Inflammatory Response Syndrome (SIRS) and Compensatory Anti-inflammatory Response Syndrome (CARS), immune cell dysfunction, and acute-phase responses.

Nutrition and inflammation are two tightly coupled pathophysiological processes that together form the core determinant of the host's ability to respond to surgical trauma. Understanding the underlying biological mechanisms is fundamental to rationally interpreting and applying related biomarkers for predicting postoperative complications, as shown in Figure 1.

### 2.1 Pathophysiological mechanisms of malnutrition

Importantly, sarcopenia should be understood as a downstream phenotype rather than a primary mechanism. It arises from chronic energy–protein deficiency, systemic inflammation, and endocrine alterations, and mediates the link between traditional nutritional indicators and postoperative outcomes (e.g., infections, delayed wound healing and prolonged recovery).

#### 2.1.1 Impact of energy and protein deficiency on tissue repair

Esophagectomy involves extensive tissue resection, digestive tract reconstruction, and anastomotic healing, a process that demands a substantial supply of energy and protein as material substrates (28). Protein-energy malnutrition (PEM) is the most common nutritional problem in ESCC patients. Protein is the raw material for synthesizing collagen, extracellular matrix, and various enzymes and structural proteins involved in cell proliferation and differentiation (29). When protein reserves (mainly skeletal muscle) are depleted, the processes of fibroblast proliferation, collagen deposition, and angiogenesis required for wound healing are severely hampered. This directly leads to poor anastomotic healing and increases the risk of anastomotic leakage—one of the most lethal complications after esophageal surgery (11, 13). Furthermore, an inadequate energy supply forces the body to break down its own tissues for fuel, exacerbating muscle and fat loss and creating a vicious cycle.

#### 2.1.2 Impact of micronutrient and vitamin deficiency on immune function

In addition to macronutrients, various micronutrients and vitamins play critical “coenzyme” or “catalyst” roles in maintaining immune homeostasis and promoting tissue repair (30, 31). For example, zinc is a component of numerous metalloenzymes and is crucial for the differentiation and function of lymphocytes; selenium is a core component of glutathione peroxidase, involved in combating oxidative stress; vitamin C is an essential cofactor for collagen synthesis; and vitamin A is vital for maintaining the integrity of mucosal barriers (32–34). Patients with ESCC often suffer from deficiencies of these micronutrients due to reduced intake and metabolic disturbances, leading to impaired function of immune cells (especially T cells and macrophages) and a decreased ability to clear pathogens, thereby significantly increasing the risk of postoperative infectious complications such as pneumonia (35–38).

### 2.2 Pathophysiological mechanisms of the systemic inflammatory response

#### 2.2.1 Tumor-Induced systemic inflammatory microenvironment

The association between chronic inflammation and the development and progression of cancer is a fundamental tenet of oncology (39–41). ESCC tumor cells and surrounding stromal cells (e.g., tumor-associated macrophages, TAMs) can continuously secrete large amounts of pro-inflammatory cytokines (e.g., IL-6, IL-8, TNF-α) and growth factors (e.g., VEGF) (42). These factors enter the systemic circulation, leading to a host systemic inflammatory response characterized by increased synthesis of acute-phase proteins (e.g., CRP), accelerated generation and mobilization of neutrophils from the bone marrow, and increased apoptosis of lymphocytes (43). This imbalanced immune state—characterized by neutrophilia and lymphopenia (reflected in indicators like an elevated NLR)—not only facilitates tumor immune evasion but also renders the body's immune response dysregulated when faced with new challenges like surgery, making it more susceptible to infection.

#### 2.2.2 The inflammatory cascade activated by surgical trauma

The major surgical trauma of esophagectomy inevitably leads to extensive tissue damage, ischemia-reperfusion injury, and endotoxin translocation, thereby triggering a violent acute inflammatory response (44). Damaged tissue cells release Damage-Associated Molecular Patterns (DAMPs), which activate Pattern Recognition Receptors (PRRs), in turn initiating an inflammatory cascade involving the complement system, coagulation system, and cytokine network (45). IL-6 is a central mediator in this process; its levels rise sharply within hours after surgery, driving the liver to synthesize CRP and other acute-phase proteins and further amplifying the inflammatory signal (46). A moderate inflammatory response is necessary for tissue repair, but an excessive or uncontrolled Systemic Inflammatory Response Syndrome (SIRS) can impair distant organ function and is a major cause of ARDS, acute kidney injury (AKI), and MODS (47).

#### 2.2.3 The role of the cytokine network in complications

Cytokines play a “double-edged sword” role in the development of postoperative complications. The excessive release of pro-inflammatory cytokines (e.g., IL-6, TNF-α, IL-1β) is the core driver of SIRS. For example, high levels of IL-6 have been shown to be associated with an increased risk of nearly all major postoperative complications after esophagectomy, including anastomotic leakage and pulmonary infections (48, 49). Concurrently, to limit the damage caused by excessive inflammation, the body compensatorily initiates an anti-inflammatory response, releasing anti-inflammatory cytokines (e.g., IL-10, TGF-β), a state known as Compensatory Anti-inflammatory Response Syndrome (CARS) (50). However, excessive CARS can lead to immunoparalysis, making the body susceptible to pathogens and serving as an important basis for secondary postoperative infections (51). Therefore, the balance between pro- and anti-inflammatory responses (the SIRS/CARS balance) determines the patient's final clinical outcome, and a dysregulation of this balance is a key mechanism for the occurrence of complications.

### 2.3 Interaction between nutrition and inflammation: the immuno-nutrition coupling mechanism

Nutrition and inflammation do not operate independently but influence each other through a complex network, jointly regulating the postoperative pathophysiological process.

#### 2.3.1 Regulation of nutritional metabolism by inflammation

A systemic inflammatory state is a key driver of cancer-related cachexia and postoperative metabolic disorders. Pro-inflammatory cytokines like IL-6 and TNF-α can directly act on the hypothalamus to cause anorexia; simultaneously, they promote skeletal muscle protein breakdown and lipolysis to provide raw materials for acute-phase protein synthesis and immune cell proliferation, but at the cost of depleting bodily reserves (52, 53). Inflammation also induces metabolic reprogramming in the liver, prioritizing the synthesis of acute-phase proteins like CRP, while the synthesis of “nutritional” proteins such as albumin and prealbumin is suppressed. Therefore, hypoalbuminemia is often not just a sign of malnutrition but also a direct reflection of the severity of the systemic inflammatory response (54).

#### 2.3.2 Impact of nutritional status on immune cell function

Conversely, nutritional status directly determines the “combat effectiveness” of the immune system. For instance, arginine is an essential amino acid for T-cell function. However, in the tumor microenvironment or under postoperative stress, arginase-1 expressed by myeloid-derived suppressor cells (MDSCs) heavily depletes arginine, leading to T-cell dysfunction (55). Omega-3 polyunsaturated fatty acids (ω-3 PUFAs), on the other hand, can serve as substrates for the production of anti-inflammatory lipid mediators (e.g., resolvins, protectins), thus helping to “extinguish” excessive inflammation (56). This provides the rationale for the perioperative use of immunonutrition formulas containing specific nutrients (e.g., arginine, ω-3 PUFAs, nucleotides), aiming to reduce postoperative complication risks by modulating the immune response (57, 58).

Beyond arginine and ω-3 PUFAs, nucleotides are conditionally essential during surgical stress, supporting lymphocyte proliferation, clonal expansion and mucosal repair; supplementation has been associated with improved lymphocyte function and barrier integrity in clinical and experimental settings (59, 60).

Glutamine fuels rapidly dividing immune cells and enterocytes and contributes to glutathione-mediated antioxidant defense. Perioperative/ICU trials suggest reduced infectious complications and shorter hospital stay with immunonutrition formulas enriched with glutamine, arginine, ω-3 and nucleotides, albeit with heterogeneity across regimens and populations (61, 62).

#### 2.3.3 The role of gut microbiota in the nutrition-inflammation-complication axis

The gut is the body's largest immune organ, and the homeostasis of the gut microbiota is crucial for maintaining local and systemic immune balance (63). Patients with ESCC often experience gut dysbiosis due to dietary changes, tumor obstruction, and antibiotic use (64). Factors such as surgery, anesthesia, and stress can further compromise the integrity of the intestinal mucosal barrier, leading to the translocation of bacteria or their products (e.g., lipopolysaccharide, LPS) into the bloodstream, which becomes a significant source for triggering or exacerbating the systemic inflammatory response (65). Nutritional status, particularly dietary fiber intake, directly influences the composition and function of the gut microbiota. A healthy microbiota can ferment fiber to produce short-chain fatty acids (SCFAs), such as butyrate, which is not only an energy source for colonocytes but also has important immunomodulatory functions like regulating Treg cell differentiation and suppressing inflammation (66). Therefore, the gut microbiota constitutes a key hub connecting nutritional intake, host immune-inflammatory status, and the risk of postoperative complications, and is emerging as a research hotspot in this field (67).

## 3 Individual nutritional and inflammatory markers for predicting postoperative complications

In clinical practice, utilizing single, easily accessible biomarkers for risk assessment is the most cost-effective and feasible approach. This chapter systematically reviews the value, limitations, and latest research progress of traditional serum nutritional markers and inflammation markers derived from blood cell counts in predicting postoperative complications in ESCC.

### 3.1 Traditional serum nutritional markers

These markers have traditionally been used to assess the body's protein reserves. However, as previously mentioned, their levels are also significantly affected by the inflammatory state, making them, in reality, a composite reflection of the “nutritional-inflammatory” status.

#### 3.1.1 Albumin (ALB)

Albumin is the most abundant protein in plasma, synthesized by the liver, with a half-life of about 21 days. It plays a key role in maintaining plasma colloid osmotic pressure and transporting various endogenous and exogenous substances (68). Preoperative hypoalbuminemia has long been considered a classic risk factor for poor surgical outcomes (69). Its predictive value stems from two mechanisms: (1) it directly reflects the long-term depletion of the body's protein reserves; and (2) as a “negative” acute-phase protein, its synthesis is inhibited by cytokines like IL-6 during a systemic inflammatory response, so low albumin levels also signify a more severe inflammatory state (70, 71).

Numerous retrospective studies and several meta-analyses have confirmed that preoperative hypoalbuminemia is an independent predictor of overall complications, severe complications (Clavien-Dindo ≥III), anastomotic leakage, and pulmonary infections after esophagectomy for ESCC (72, 73). A 2022 meta-analysis including over 15,000 esophageal cancer patients indicated that for every 10 g/L decrease in preoperative albumin, the risk of postoperative complications increases by about 1.6-fold (74). Despite its established predictive value, the limitations of albumin are also significant: its long half-life prevents it from sensitively reflecting short-term changes in nutritional status, and its levels are easily influenced by various non-nutritional factors such as liver function, renal function, and hydration status (75, 76).

#### 3.1.2 Prealbumin (PALB)

Prealbumin (also known as transthyretin) is also synthesized by the liver, but its half-life is only 2–3 days, making it a more sensitive indicator of acute nutritional changes than albumin (77, 78). In theory, PALB can reveal deterioration or improvement in nutritional status earlier. Several studies have shown that low preoperative PALB levels are associated with an increased risk of postoperative infectious complications, anastomotic leakage, and overall complications in ESCC (79, 80). A study on ESCC patients receiving neoadjuvant chemotherapy found that the decline in PALB levels after treatment, rather than the absolute value, was more effective in predicting postoperative pulmonary complications (81). However, similar to albumin, PALB is also a negative acute-phase protein, and its level drops rapidly under acute stress and inflammation, making it difficult to interpret solely as a nutritional marker in an inflammatory context (82, 83).

#### 3.1.3 Transferrin (TRF) and retinol-binding protein (RBP)

TRF (half-life ∼8 days) and RBP (half-life only 12 hours) have also been studied as nutritional assessment markers. Their half-lives are intermediate (TRF) or extremely short (RBP), theoretically offering different values for dynamic monitoring (84). Some small-scale studies have explored their relationship with postoperative complications in ESCC, but the results are not as consistent or robust as those for ALB and PALB (85). Currently, due to issues with testing availability, cost, and susceptibility to specific factors like iron metabolism (TRF) and vitamin A levels (RBP), their application in routine clinical risk assessment is far less common than that of albumin and prealbumin.

### 3.2 Inflammation markers based on blood cell counts

A Complete Blood Count (CBC) is one of the most routine tests for all hospitalized patients. Various ratios derived from it can conveniently and dynamically reflect changes in the counts of key immune cells such as neutrophils, lymphocytes, monocytes, and platelets, thereby quantifying the intensity of the systemic inflammatory response and the state of immunosuppression.

#### 3.2.1 C-reactive protein (CRP)

CRP is a classic acute-phase protein synthesized by the liver under the stimulation of pro-inflammatory cytokines like IL-6, and it serves as the “gold standard” for measuring acute inflammation and tissue injury (86). Elevated preoperative CRP reflects a chronic inflammatory state driven by the tumor itself and has been proven to be an adverse prognostic factor in various cancers. In ESCC, high preoperative CRP levels are significantly associated with a higher incidence of postoperative complications, especially infectious complications and anastomotic leakage (87, 88).

Of even greater value is the dynamic monitoring of postoperative CRP changes. After esophagectomy, CRP levels typically peak on postoperative day 2–3 and then gradually decline. If CRP levels fail to decrease or rise again between postoperative days 3–5, it strongly suggests the occurrence of complications, particularly anastomotic leakage and infection (80). One study showed that a CRP level >170 mg/L on postoperative day 4 is a highly effective indicator for predicting anastomotic leakage, with a very high negative predictive value, aiding in safe clinical decision-making (e.g., early removal of drains or initiation of oral intake) (89).

#### 3.2.2 Neutrophil-to-lymphocyte ratio (NLR)

The NLR has been one of the most prominent markers in tumor immunology research in recent years. It cleverly integrates information from two major types of immune cells: neutrophils (reflecting pro-tumor inflammation) and lymphocytes (reflecting anti-tumor immunity). Tumor-related inflammation stimulates the bone marrow to release large numbers of neutrophils, while the tumor microenvironment and systemic cytokines induce lymphocyte apoptosis, leading to an elevated NLR (90). Therefore, a high NLR represents an imbalance towards a “pro-inflammatory/immunosuppressive” state.

Numerous retrospective studies and meta-analyses have consistently shown that a high preoperative NLR is a powerful independent predictor of postoperative complications (including overall complications, pulmonary complications, anastomotic leakage, cardiovascular events) and short-term mortality in ESCC patients (91, 92). A key clinical challenge is the lack of a standardized optimal cut-off value, which ranges from 2.0 to 5.0, limiting direct comparisons across different studies and institutions (93). Furthermore, dynamic changes in postoperative NLR also have predictive value, with a sustained elevation or a “second peak” often serving as an early warning sign of complications (94).

#### 3.2.3 Platelet-to-lymphocyte ratio (PLR)

Platelets not only participate in hemostasis but also play an active role in inflammation and tumor progression, capable of secreting various pro-inflammatory and pro-angiogenic factors (95). Similar to the NLR, the PLR integrates information on both pro-inflammatory (thrombocytosis) and immunosuppressive (lymphopenia) aspects. Several studies have confirmed that an elevated preoperative PLR is associated with an increased risk of postoperative complications and shorter survival in ESCC, although its predictive efficacy is generally considered slightly inferior to that of the NLR (96, 97). Several studies have adopted composite indices such as the Systemic Immune-Inflammation Index (SII = platelet × neutrophil/lymphocyte), which integrates PLR and NLR components and outperforms either marker alone in predicting postoperative outcomes and survival in ESCC cohorts (98, 99).

#### 3.2.4 Lymphocyte-to-monocyte ratio (LMR)

The LMR is another indicator reflecting immune balance. Lymphocytes represent adaptive immunity, while monocytes can differentiate into pro-tumor M2-type macrophages. Therefore, a low LMR may reflect weakened adaptive immune surveillance and a dominance of myeloid-derived pro-tumor cells (100). In ESCC patients, a low preoperative LMR has been found to be associated with a higher rate of postoperative complications and poorer prognosis (101). However, research on LMR is less extensive compared to NLR and PLR, and its independent predictive value still requires validation in more large-scale studies.

#### 3.2.5 Systemic immune-inflammation index (SII)

To integrate more dimensions of immune-inflammatory information, the SII was proposed, calculated as: SII = (Platelet count × Neutrophil count)/Lymphocyte count (92). This index simultaneously considers the changes in platelets, neutrophils, and lymphocytes, theoretically providing a more comprehensive reflection of the body's inflammatory and immune status. In recent years, the value of SII in predicting the prognosis of ESCC has gained increasing attention. Multiple studies have shown that a high preoperative SII is a potent predictor of postoperative complications and poor long-term survival, with its predictive efficacy potentially superior to that of NLR or PLR alone (93, 94).

## 4 Combined nutritional and inflammatory scoring systems and their predictive value

Given the individual limitations of single markers, researchers have developed a series of composite scoring systems by integrating multiple nutritional and inflammatory indicators, aiming to improve predictive accuracy and robustness.

### 4.1 Prognostic nutritional index (PNI)

The PNI is one of the earliest and most widely used nutritional-immune assessment tools, first proposed by the Japanese scholar Onodera in 1984 for gastrointestinal surgery patients. It is calculated as: PNI = Serum albumin (g/L) + 5 × Total peripheral lymphocyte count (10^9^/L) (95). The PNI cleverly combines albumin, which reflects long-term nutritional status and chronic inflammation, with lymphocytes, which represent cellular immune status.

In the field of ESCC, the predictive value of PNI has been repeatedly validated. Numerous retrospective studies and meta-analyses have confirmed that a low preoperative PNI is an independent risk factor for overall complications, severe complications, pulmonary infections, and anastomotic leakage after esophagectomy (96, 97). A 2023 study comparing various nutritional scores for predicting postoperative complications after neoadjuvant therapy in ESCC found that the predictive efficacy of PNI was superior to most other indicators (98). The advantages of PNI lie in its simple calculation and readily available parameters, making it highly practical in the clinical setting.

### 4.2 Glasgow prognostic score (GPS) and its modified version (mGPS)

The GPS and its modified version (mGPS) are purely inflammation-based scoring systems that combine C-reactive protein (reflecting acute inflammation) and albumin (reflecting chronic inflammation and nutrition). The mGPS is scored as follows: CRP ≤ 10 mg/L receives 0 points; CRP > 10 mg/L receives 1 point; CRP > 10 mg/L and albumin <35 g/L receives 2 points (99).

The mGPS has been proven to be a powerful prognostic tool in various solid tumors. In ESCC patients, those with a preoperative mGPS of 1 or 2 have a significantly higher incidence of postoperative complications than those with a score of 0 (100, 101). The advantage of mGPS is that it bypasses the variability of lymphocyte counts and integrates both acute and chronic inflammatory markers. Some studies suggest that mGPS is stronger at predicting long-term survival than short-term complications (102).

### 4.3 Controlling nutritional status (CONUT) score

CONUT scoring: albumin (≥3.5 g/dl = 0; 3.0–3.49 = 2; 2.5–2.99 = 4; < 2.5 = 6), total lymphocyte count (≥1,600/µl = 0; 1,200–1,599 = 1; 800–1,199 = 2; <800 = 3), and total cholesterol (≥180 mg/dl = 0; 140–179 = 1; 100–139 = 2; <100 = 3). Risk strata: normal 0–1; mild 2–4; moderate 5–8; severe 9–12. A stepwise rise in postoperative complications has been reported with increasing categories in ESCC surgical cohorts (103). This scoring system not only considers protein reserves and immune status but also incorporates lipid metabolism, making it theoretically more comprehensive. Based on the levels of these three indicators, patients are classified as having normal, mild, moderate, or severe malnutrition. In ESCC patients, an elevated preoperative CONUT score (indicating poorer nutritional status) is associated with a higher risk of postoperative complications (104, 105). The predictive ability of CONUT is comparable to that of PNI, but the inclusion of cholesterol makes its underlying biological interpretation more complex.

### 4.4 NLR-based and albumin-based scores (e.g., NLR-albumin score)

To combine the strong inflammatory predictive power of NLR with the classic nutritional assessment value of albumin, new combination scores have been proposed. For example, one study created a simple 0-1-2 point system by treating high NLR and low albumin as risk factors (0 points for no risk factors, 1 for one, 2 for both). This simple combination proved effective in stratifying the risk of postoperative complications in ESCC, with its predictive ability superior to that of NLR or albumin alone (106).

### 4.5 Other emerging composite markers

As research deepens, more novel composite markers are emerging to identify better predictors. C-reactive protein/albumin ratio (CAR) captures the balance between systemic inflammation and nutritional reserve; higher CAR predicts infectious morbidity and poorer survival after ESCC resection. Fibrinogen-to-albumin ratio (FAR) integrates pro-coagulant inflammatory tone with protein reserve; elevated FAR correlates with increased postoperative complications. SII (platelet × neutrophil/lymphocyte) frequently outperforms NLR or PLR alone for prognosis. Cut-offs vary across studies; prospective validation is needed before standardization (107–109).

### 4.6 Comparative specificity of different markers for different complications (e.g., anastomotic leakage, pulmonary infection)

An important clinical question is whether specific markers have higher predictive specificity for particular complications. Existing evidence suggests such a trend may exist.

#### 4.6.1 Anastomotic leakage

Directly related to inflammation and tissue healing. Therefore, indicators reflecting the intensity of acute inflammation (e.g., high or persistently elevated postoperative CRP) and those reflecting tissue repair capacity (e.g., low preoperative albumin, low PNI) are considered to have strong predictive value (81, 97).

#### 4.6.2 Pulmonary infection

Closely related to immunosuppression and systemic inflammation. Thus, indicators reflecting an imbalance in immune cells (e.g., high NLR, high SII, low PNI) show strong predictive ability (84, 94). Sarcopenia, as a morphological indicator leading to respiratory muscle weakness and difficulty in expectoration, is particularly closely associated with pulmonary complications (39).

However, it must be emphasized that the occurrence of most complications is the result of multiple factors. Therefore, it is unrealistic to expect a single marker to perfectly predict a specific complication. The current consensus is that composite markers or multivariate prediction models (such as the nomograms discussed in the next chapter) that integrate information from multiple dimensions generally have superior overall predictive performance. Table 1 offers a comprehensive summary of these key biomarkers, detailing their calculation methods, common clinical thresholds, and their primary applications in predicting postoperative complications.

**Table 1:** Summary of nutritional and inflammatory biomarkers for predicting postoperative complications in ESCC.

Biomarker category	Biomarker name	Calculation/formula	Units	Typical cut-off value(s)	Primary predicted complications	Key references
Nutritional markers	Albumin (ALB)	Direct serum measurement	g/L	< 35–40 (Hypoalbuminemia)	Overall complications, severe complications, anastomotic leakage, pulmonary infection	(65–68)
	Prealbumin (PALB)	Direct serum measurement	mg/L	Low levels; dynamic decline is also significant	Infectious complications, anastomotic leakage, overall complications	(71–73)
Inflammatory markers	C-reactive protein (CRP) (mg/L)	Direct serum measurement	mg/L	Preoperative: >10; Postoperative: Peak on POD 2–3, failure to decline by POD 4–5	Anastomotic leakage, infectious complications, overall complications	(78–81, 99)
	Neutrophil-to-lymphocyte ratio (NLR)	Neutrophil count/lymphocyte count	^a^	High (e.g., >2.0–5.0)	Pulmonary complications, anastomotic leakage, cardiovascular events, overall complications	(83–86)
	Platelet-to-lymphocyte ratio (PLR)	Platelet count/lymphocyte count	^a^	High	Overall complications	(88, 89)
	Systemic immune-inflammation index (SII)	(Platelet × neutrophil)/lymphocyte	^a^	High (e.g., >900)	Overall complications (potentially superior to NLR/PLR)	(93, 94)
	Lymphocyte-to-monocyte ratio (LMR)	Lymphocyte count/monocyte count	^a^	Low	Overall complications	(91)
Combined scores	Prognostic nutritional index (PNI)	Albumin (g/L) + 5 × lymphocyte count (10⁹/L)	^a^	Low (e.g., <45)	Severe complications, pulmonary infections, anastomotic leakage, overall complications	(95–98)
	Modified glasgow prognostic score (mGPS)	Score (0–2) based on CRP (>10 mg/L) and albumin (<35 g/L)	^a^	Score 1 or 2	Overall complications, long-term survival	(99–102)
	Controlling nutritional status (CONUT) score	Score based on albumin, lymphocyte count, and cholesterol	^a^	High score indicates higher risk	Overall complications	(104, 105)
	C-reactive protein to albumin ratio (CAR)	CRP (mg/L)/albumin (g/L)	^a^	High	Overall complications	(108, 109)
	Fibrinogen-to-albumin ratio (FAR)	Fibrinogen (g/L)/albumin (g/L)	^a^	High	Overall complications	(107)

The cut-off values for these biomarkers are not universally standardized and can vary significantly across different studies, patient populations, and institutions. The values listed represent commonly cited thresholds for defining “high-risk” groups in the literature.

a Means unitless index.

## 5 Clinical applications: integrating markers into perioperative patient management

The ultimate goal of a deep understanding of nutritional and inflammatory markers is to effectively integrate them into the clinical decision-making process of perioperative management, shifting from “reactive treatment” of complications to “proactive prevention.” This requires a systematic strategy covering the preoperative, intraoperative, and postoperative periods.

### 5.1 Preoperative risk stratification and identification of high-risk patients

Accurate preoperative risk stratification is the prerequisite for implementing individualized perioperative management.

#### 5.1.1 Building and validating prediction models, with nomograms as graphical representations

**Figure 2:**
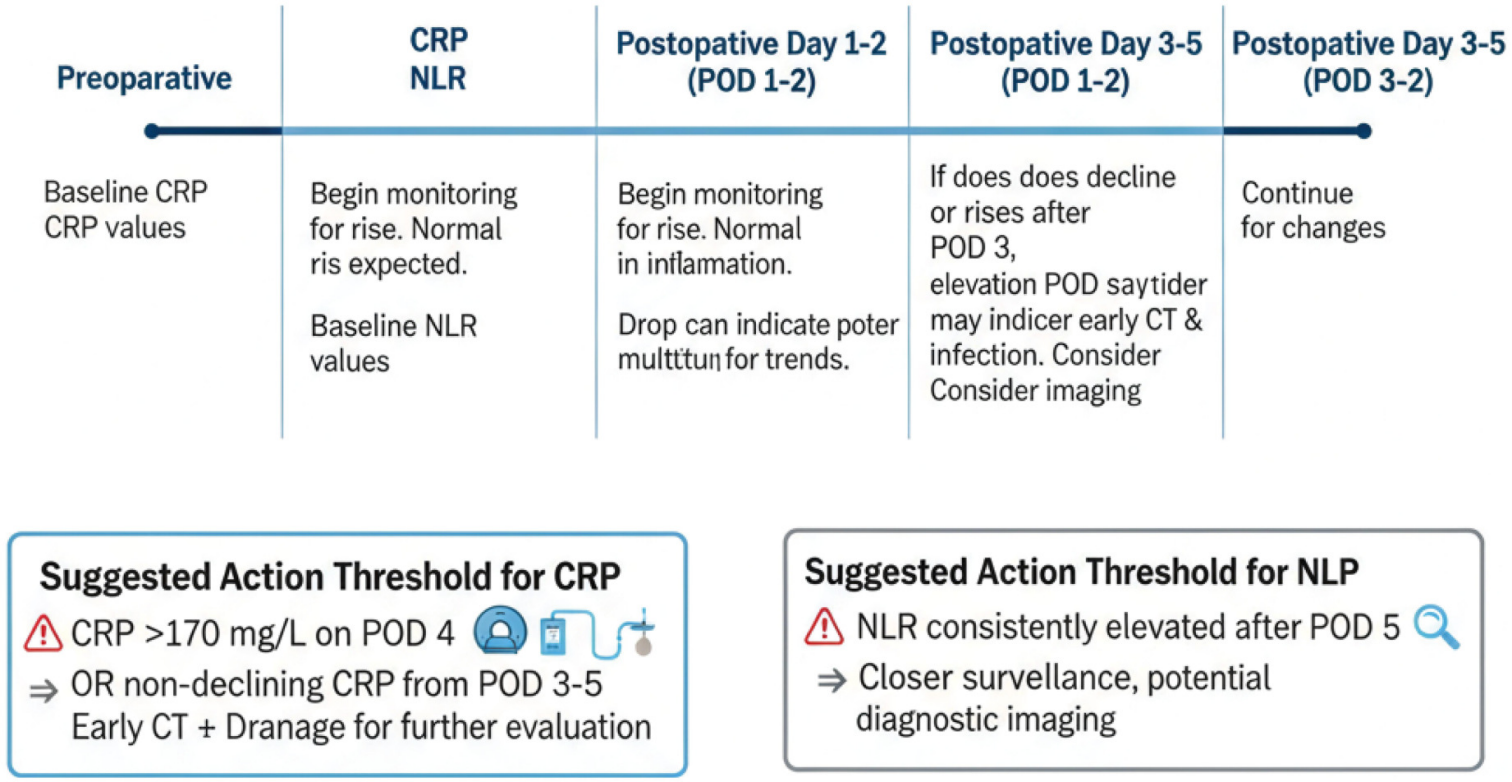
Workflow of biomarker-based prediction in ESCC. This flowchart outlines the steps in biomarker-based prediction of postoperative complications: (1) variable selection, (2) model building (using regression or machine learning approaches), (3) internal and external validation (including AUC, calibration, and decision curve analysis), (4) visualizing the prediction model as a nomogram, and (5) integrating the nomogram into clinical decision-making.

A nominal nomogram is not a prediction model itself but a graphical representation of an underlying statistical or machine-learning model (e.g., logistic/Cox regression, random forest) (110). The model must first be specified, internally and externally validated (discrimination, calibration, decision-curve analysis), and only then translated into a nomogram for clinical use. As shown in Figure 2, we therefore outline a stepwise framework: variable selection → model development → internal/external validation → visualisation as a nomogram → clinical integration (111).

In recent years, several studies have developed nomograms specifically for predicting postoperative complications in ESCC patients. For instance, one study integrated mGPS, age, and surgical approach to build a model for predicting major complications, which showed good calibration and discrimination (C-index >0.80) (112). Another study combined PNI, sarcopenia status, and pulmonary function indicators to create a nomogram for predicting postoperative pulmonary complications, with its predictive performance significantly superior to any single indicator (113). The development and internal/external validation of these models provide powerful tools for clinicians to assess patient risk preoperatively and to communicate effectively with patients and their families.

#### 5.1.2 Multimodal prediction by incorporating imaging features (e.g., CT-measured muscle mass)

Combining blood biomarkers with imaging information is another important avenue for achieving more precise risk stratification. As mentioned earlier, sarcopenia, diagnosed by measuring the skeletal muscle index (SMI) at the L3 level on preoperative CT scans, is a powerful predictor of poor outcomes after esophagectomy for ESCC (39). Sarcopenia reflects the “morphological” depletion of the body's protein reserves, while blood markers reflect the “functional” state of nutrition and inflammation. The two provide complementary information. Studies have shown that patients with both sarcopenia and a high NLR (or low PNI) have a manifold increase in the risk of postoperative complications, constituting an extremely high-risk subgroup (114, 115). Incorporating SMI as a continuous variable into the aforementioned nomogram models has been shown to further enhance their predictive efficacy. Future models may even integrate more complex radiomics features to uncover additional prognostic information from imaging (116).

### 5.2 Guiding perioperative nutritional support strategies

After identifying high-risk patients, the next key step is to take targeted interventions, with perioperative nutritional support being a core component.

#### 5.2.1 Evidence and application of preoperative immunonutrition

For patients identified as being at nutritional risk through markers (e.g., low PNI, low albumin) or scoring systems (e.g., NRS2002, MUST), preoperative nutritional intervention has become a consensus. In recent years, the concept of “immunonutrition” has gained considerable attention. Immunonutrition formulas typically refer to enteral nutrition preparations fortified with specific immunomodulatory nutrients (e.g., arginine, ω-3 polyunsaturated fatty acids, nucleotides) (57). The rationale is to enhance the body's tolerance to surgical trauma by modulating the preoperative immune-inflammatory state.

The European Society for Clinical Nutrition and Metabolism (ESPEN) guidelines recommend that patients undergoing major upper gastrointestinal cancer surgery should routinely receive oral immunonutrition for 5–7 days preoperatively, regardless of their preoperative nutritional status (117). Several meta-analyses have confirmed that the perioperative use of immunonutrition can significantly reduce the incidence of infectious complications and shorten the length of hospital stay after surgery for upper gastrointestinal cancers, including esophageal cancer (118, 119). Therefore, patients identified with a high inflammatory state using markers like PNI or GPS may be the primary beneficiaries of immunonutrition. Future research should shift from a “one-size-fits-all” application to “precision” immunonutrition guided by biomarkers.

#### 5.2.2 Selection and monitoring of postoperative nutritional routes

Early and adequate postoperative nutritional support is crucial for compensating for surgical catabolism and promoting anastomotic healing. The traditional “nil per os + parenteral nutrition” model has been shown to increase infection risk and lead to atrophy of the intestinal barrier function. Early enteral nutrition (EEN) has become standard practice (117). However, for patients identified preoperatively with severe malnutrition or a high inflammatory state, a more aggressive nutritional strategy may be required, such as early combination with parenteral nutrition (PN) to ensure adequate energy and protein supply, or more cautious selection of feeding tube routes to balance the benefits of enteral nutrition with the risk of aspiration (120). Furthermore, dynamic postoperative monitoring of short-half-life proteins like prealbumin (PALB) can help assess the effectiveness of nutritional support and allow for timely adjustments to the nutritional plan (73).

### 5.3 Dynamic monitoring of markers to predict early complications

Early diagnosis of postoperative complications, especially anastomotic leakage, is often difficult, and delayed diagnosis can have severe consequences. Dynamic monitoring of the trajectory of inflammatory markers offers a potential for early warning.

#### 5.3.1 Dynamic trajectory of postoperative CRP, NLR, and other indicators

**Figure 3:**
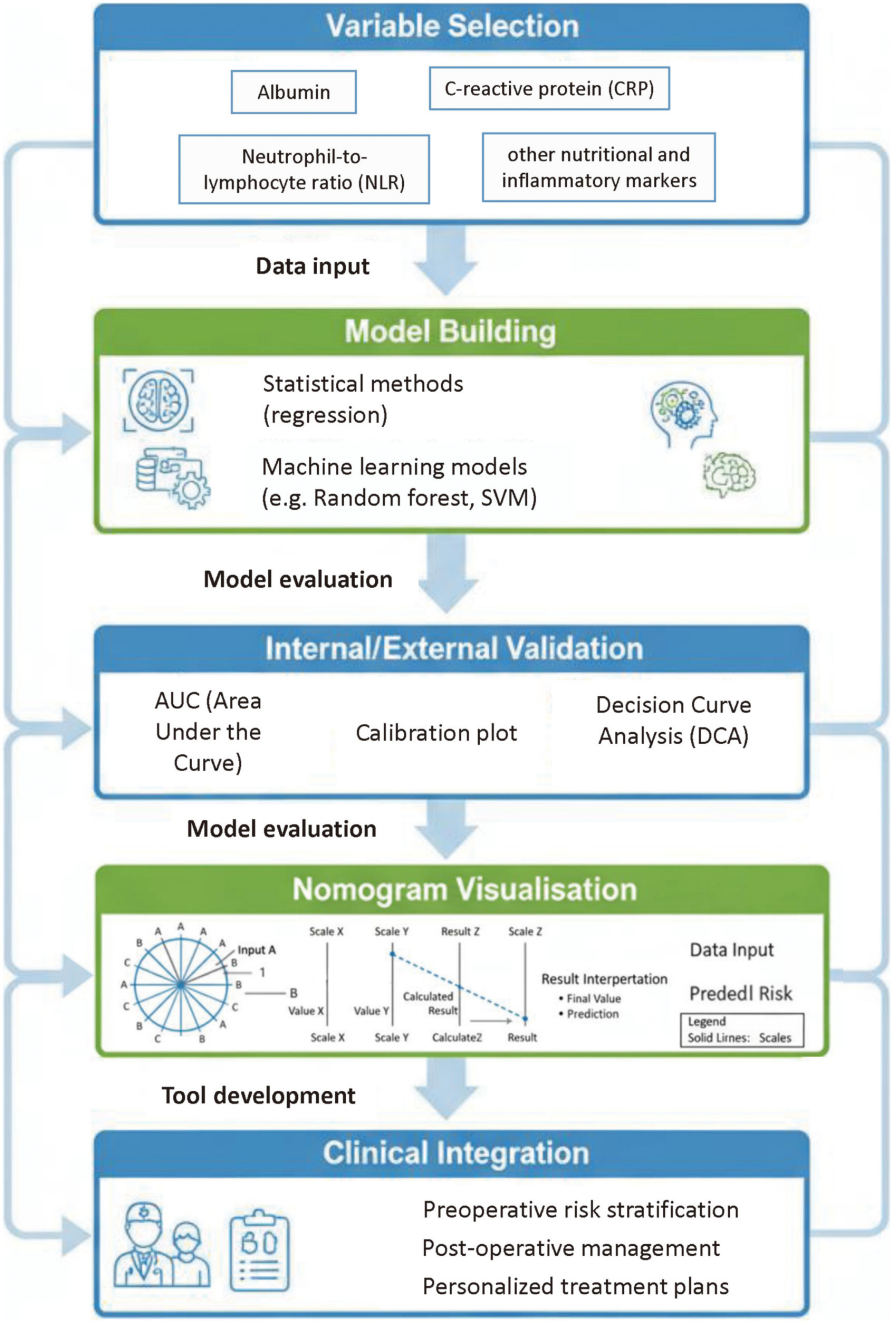
Perioperative monitoring timeline for CRP, NLR, and PNI with suggested action thresholds. This timeline depicts the perioperative monitoring of C-reactive protein (CRP), neutrophil-to-lymphocyte ratio (NLR), and Prognostic Nutritional Index (PNI), with suggested action thresholds. For example, a CRP level >170 mg/L on postoperative day 4 (POD4) or a non-declining CRP from POD3–5 indicates the need for early imaging (e.g., contrast-enhanced CT) and possible drainage intervention.

As detailed in Section 3.2.1, CRP typically peaks on POD 2–3. For clinical use, we recommend that persistently high or re-rising CRP by POD 3–5 trigger early imaging (e.g., contrast-enhanced CT) and source control when appropriate, as shown in Figure 3 and Table 2. Similarly, sustained postoperative NLR elevation should prompt closer surveillance and targeted diagnostics (81, 86, 121).

**Table 2:** Actionable thresholds for key biomarkers/composite scores and recommended clinical responses.

Marker/parameter	Cut-off	Associated risk	Suggested action
CRP (C-reactive protein)	POD4 > 170 mg/L	High risk of anastomotic leakage (AL)	High suspicion for AL → CT scan + consider drainage/endoscopy.
NLR (neutrophil-to-lymphocyte ratio)	Sustained elevation	Infection risk	Early warning for infection → increase monitoring/targeted diagnostics.
CONUT (controlling nutritional status)	5–8 (moderate malnutrition), 9–12 (severe malnutrition)	Moderate to severe malnutrition	Preoperative immunonutrition ± postoperative EN + PN.
Albumin (ALB)	< 3.0 g/dl	Poor nutritional reserves, risk for complications	Nutritional support → enteral/parenteral nutrition.
Prealbumin (PALB)	< 10 mg/dl	Acute malnutrition risk	Preoperative nutritional support + close monitoring for infectious complications.
SII (systemic immune-inflammation index)	High (>900)	Severe systemic inflammation	Close postoperative monitoring + early intervention for infection management.

#### 5.3.2 “Second hit” theory and complication warning

The “second hit” theory underpins this clinical phenomenon (22). The preoperative tumor burden and malnutrition constitute the “first hit,” placing the body in a fragile, compensated state of inflammatory/anti-inflammatory imbalance. Surgical trauma acts as the “second hit,” completely disrupting this unstable equilibrium and leading to uncontrolled inflammation and immunosuppression. A subsequent postoperative complication (e.g., intra-abdominal infection from an anastomotic leak) may constitute a “third hit,” ultimately leading to MODS and death. Dynamic monitoring of markers like CRP and NLR is, in effect, a real-time “quantification” of the intensity and direction of the body's inflammatory response after each hit, thus providing a warning before a clinical catastrophe occurs.

## 6 Future perspectives

Although existing nutritional and inflammatory markers have shown great clinical potential, there is still ample room for exploration in this field. Future developments will focus on discovering novel biomarkers, applying more powerful analytical tools, and designing more precise intervention strategies.

### 6.1 Exploration of novel biomarkers

#### 6.1.1 Application of metabolomics and proteomics

Traditional blood markers represent only the tip of the iceberg of a complex pathophysiological network. High-throughput “omics” technologies, such as metabolomics and proteomics, can simultaneously detect hundreds or thousands of metabolites and proteins in blood or tissue samples, providing unprecedented depth and breadth to profile a patient's “molecular phenotype” (122). Preliminary studies have already attempted to use these technologies to find new targets for predicting surgical complications. For example, by analyzing the preoperative plasma metabolome, changes in specific amino acids (e.g., branched-chain amino acids) and lipid molecules have been found to be associated with the risk of postoperative infection (123). Proteomics has the potential to discover novel inflammation- or nutrition-related protein markers that are more specific and sensitive than CRP or albumin. The challenges of these technologies include high costs, complex data analysis, and the need for validation in large-scale samples.

#### 6.1.2 Circulating tumor DNA (ctDNA) and minimal residual disease (MRD)

Cell-free DNA (cfDNA) arises from both normal and malignant cells via apoptosis, necrosis or active secretion; ctDNA is the tumour-derived fraction of cfDNA carrying somatic mutations/methylation signals. Surgical trauma can transiently elevate total cfDNA, necessitating careful timing and serial sampling. ctDNA-based MRD detection after esophagectomy correlates with early relapse; integrating cfDNA/ctDNA dynamics with inflammatory markers (CRP/NLR) may refine early risk stratification and guide surveillance or adjuvant strategies (124, 125). ctDNA consists of DNA fragments released into the bloodstream from tumors. The detection of postoperative ctDNA (i.e., minimal residual disease, MRD) has been proven to be an ultra-early predictor of recurrence for various cancers (124). Although ctDNA is primarily used for monitoring tumor burden and recurrence risk, its relationship with perioperative complications is also worth exploring. A patient with a high tumor burden releasing large amounts of ctDNA may also have a more severe systemic inflammatory state. Investigating the association between preoperative ctDNA levels or postoperative ctDNA clearance dynamics and the risk of complications is an interesting new direction (125).

#### 6.1.3 Monitoring of specific immune cell subsets

Indicators like NLR and PLR only reflect crude changes in immune cell numbers. Techniques such as flow cytometry allow for more refined typing and functional analysis of immune cells. For example, myeloid-derived suppressor cells (MDSCs) and regulatory T cells (Tregs) are two key types of immunosuppressive cells in the tumor microenvironment and systemic circulation (55). Studies have already shown that high levels of peripheral MDSCs before surgery are associated with a poor prognosis in esophageal cancer patients (126). Monitoring the quantity and functional changes of these specific immune cell subsets could provide a more precise assessment of immune status than NLR, thereby more accurately predicting the risk of infectious complications.

### 6.2 Application of artificial intelligence and machine learning in prediction model construction

Faced with an increasing amount of multidimensional data (clinical, biochemical, imaging, omics), traditional statistical methods (e.g., logistic regression) may struggle to capture the complex nonlinear relationships within. Artificial intelligence (AI), particularly machine learning (ML), offers a powerful solution (127).

#### 6.2.1 Deep learning models for integrating multidimensional data

Machine learning algorithms, such as random forests, support vector machines, and neural networks, can handle high-dimensional data and autonomously learn and identify complex patterns from it. For example, an ML model could be developed that simultaneously inputs a patient's demographic information, blood markers, CT radiomics features, and even genomic data to generate a highly individualized prediction of complication risk (128). Deep learning models are particularly powerful in processing image data and may, in the future, enable the automated extraction of information like sarcopenia and visceral adiposity from CT scans, seamlessly integrating it with blood markers to build “end-to-end” intelligent prediction systems.

#### 6.2.2 Personalized, dynamic risk prediction systems

Future risk prediction systems will no longer be static. By integrating continuously monitored vital signs and dynamically changing biomarker data (e.g., CRP, NLR every 12 hours), time-series analysis algorithms (e.g., recurrent neural networks, RNNs) can be used to build a dynamic, real-time updated risk warning system. When the system predicts that a patient's risk of complications exceeds a certain threshold, it could automatically alert the clinical team, truly achieving “smart healthcare” (129).

### 6.3 Marker-guided precision intervention and clinical trial design

#### 6.3.1 “Basket trials” for validating different interventions

Future clinical trial designs need to be more precise. The “basket trial” model from oncology drug development can be adapted. For example, a trial could be designed to enroll all high-risk ESCC patients identified preoperatively by biomarkers (e.g., PNI < 45 or SII > 900), who are then randomized into different intervention “baskets,” such as: (A) standard perioperative care; (B) preoperative enhanced immunonutrition; (C) preoperative rehabilitation (“prehabilitation”); (D) combined immunonutrition + prehabilitation. This design can efficiently validate the effectiveness of different interventions in a specific high-risk population (130).

#### 6.3.2 Establishing international, multi-center, standardized databases and prospective studies

The vast majority of current research in this field consists of single-center retrospective analyses, which suffer from selection bias and non-uniform cut-off values. Future breakthroughs urgently require large-scale, multi-center, prospective cohort studies. Establishing international, standardized perioperative databases for ESCC, with uniform standards for data collection, marker measurement, and complication definitions [e.g., using the Esophagectomy Complications Consensus Group (ECCG) criteria], is crucial for developing and validating universally applicable prediction models and intervention strategies (9).

### 6.4 Challenges and opportunities for clinical translation

Translating these research findings into daily clinical practice still faces challenges, including how to standardize testing and reporting, how to determine universally applicable cut-off values, how to integrate complex models into busy clinical workflows, and the cost-effectiveness of novel markers and technologies. However, the opportunities are also immense. By more intelligently utilizing these inexpensive and readily available blood markers and embracing new technologies, we have the potential to significantly improve our ability to identify high-risk patients and, through precision intervention, ultimately improve the clinical outcomes for ESCC patients undergoing esophagectomy—one of the core tenets of the precision surgery era.

## 7 Conclusion

Esophagectomy is the cornerstone of treatment for localized esophageal squamous cell carcinoma, but the persistently high rate of postoperative complications severely impacts patients’ short-term recovery and long-term survival. The host's nutritional status and systemic inflammatory response are key intrinsic factors that determine the body's tolerance to surgical trauma and its capacity for repair. These two factors are intertwined and together form the pathophysiological basis for the development of complications.

This review has systematically summarized the recent progress in using nutrition- and inflammation-related biomarkers to predict postoperative complications in ESCC. We have elucidated how malnutrition (especially protein-energy malnutrition and sarcopenia) and systemic inflammation (driven by both the tumor and surgical trauma) create conditions for complications by impairing tissue repair, weakening immune function, and exacerbating catabolism.

Clinically, a series of easily accessible and low-cost blood biomarkers—from classic markers like albumin and prealbumin, to widely studied inflammatory indices like NLR, PLR, and SII, and composite scoring systems such as PNI, mGPS, and CONUT—have been repeatedly proven to have significant predictive value. They can effectively stratify patients by risk before surgery, identifying those most likely to benefit from enhanced perioperative interventions like immunonutrition. Furthermore, dynamic postoperative monitoring of markers such as CRP and NLR provides a powerful tool for the early warning and diagnosis of severe complications like anastomotic leakage.

Looking ahead, the field is moving towards deeper, broader, and more intelligent approaches. The exploration of novel biomarkers through metabolomics and proteomics, the construction of AI-based prediction models integrating multimodal data, and the design of biomarker-guided precision intervention clinical trials will be the focus of future research. Integrating these advanced concepts and tools into clinical practice holds the promise of ultimately achieving individualized and precise perioperative management for ESCC patients, thereby minimizing complication risks and improving their overall prognosis.

## Data Availability

The datasets presented in this study can be found in online repositories. The names of the repository/repositories and accession number(s) can be found in the article/Supplementary Material.
